# Coexistence of a novel *WISP3* pathogenic variant and an *MEFV* mutation in an Arabic family with progressive pseudorheumatoid dysplasia mimicking polyarticular juvenile idiopathic arthritis

**DOI:** 10.1186/s12969-020-00462-5

**Published:** 2020-09-07

**Authors:** Basil M. Fathalla, Elham Ahmed Elgabaly, Ahmad Abou Tayoun

**Affiliations:** 1 Section of Pediatric Rheumatology, Department of Medical Subspecialties, Al Jalila Children’s Specialty Hospital, Dubai, United Arab Emirates; 2 Department of Radiology, Al Jalila Children’s Specialty Hospital, Dubai, United Arab Emirates; 3 Department of Genomics, Al Jalila Children’s Specialty Hospital, Dubai, United Arab Emirates

**Keywords:** Progressive pseudorheumatoid dysplasia, Juvenile idiopathic arthritis, Familial Mediterranean fever, Whole-exome sequencing

## Abstract

**Background:**

A spectrum of rare noninflammatory disorders may present with arthropathy that arises from bony dysplasia, a thickened synovium, and noninflammatory effusion, leading to a constellation of clinical features that mimics chronic polyarticular juvenile idiopathic arthritis (JIA). We report a unique Arabic family harboring a novel pathogenic variant in the *WISP3* gene and presenting with progressive pseudorheumatoid dysplasia (PPRD), a rare noninflammatory arthropathy mimicking polyarticular JIA.

**Case presentation:**

An Arabic family with PPRD was diagnosed using whole-exome sequencing (WES), revealing a novel c.707delG pathogenic variant in the *WISP3* gene. The proband was referred at 10 years old for possible diagnosis of polyarticular JIA based on progressive arthropathy for three years. He was already on naproxen and methotrexate. We suspected familial noninflammatory arthropathy based on clinical manifestations, imaging findings, and family history. WES confirmed the molecular diagnosis of PPRD in the proband and one sister with a similar phenotype. An unexpected p.A744S *MEFV* pathogenic variant was detected in the proband, parents, and affected sister.

**Conclusions:**

Early identification and diagnosis of familial noninflammatory arthropathies such as PPRD can prevent unnecessary use of immunosuppressive medications. Diagnosis requires high suspicion in children with early onset arthritic changes, absence of elevated inflammatory markers, specific imaging findings, and positive family history suggestive of an autosomal recessive disorder. We highlight the advantages of WES over single-gene analysis in such cases.

## Background

Juvenile idiopathic arthritis (JIA) represents a heterogeneous group of relatively common chronic arthritides in children [1]. A spectrum of rare noninflammatory disorders may present with arthropathy that arises from bony dysplasia, a thickened synovium, and noninflammatory effusion, leading to clinical manifestations that often mimic JIA [2, 3]. Progressive pseudorheumatoid dysplasia (PPRD) is an autosomal recessive noninflammatory arthropathy (OMIM #208230) characterized by progressive joint stiffness, joint swelling with contractures, and a waddling gait. The clinical manifestations may mimic polyarticular juvenile idiopathic arthritis (poly-JIA), especially in the early phase of the disease, delaying an accurate diagnosis and leading to unnecessary use of immunosuppressive medications [3, 4]. PPRD has a progressive disease course leading to significant disability with no known treatment; in contrast, recent advances in the treatment of poly-JIA have yielded significantly improved outcomes [5]. Accordingly, differentiating both entities has a significant impact on patient management and outcomes.

We report a case of a child referred to our center with chronic arthropathy mimicking poly-JIA. The patient was already on naproxen and methotrexate. However, we suspected PPRD based on the clinical and imaging findings as well as family history. Whole-exome sequencing (WES) revealed a WNT1-inducible signaling pathway protein 3 (*WISP3*) homozygous mutation along with an unexpected *MEFV* mutation in the family. With this report, we aim to increase awareness about PPRD and other rare noninflammatory arthropathies as conditions that can mimic poly-JIA and highlight the key clinical and radiological aspects of PPRD. This will aid clinicians, enable an early diagnosis and prevent unnecessary use of immunosuppressive medications. We also specifically discuss the role of WES in investigating familial noninflammatory arthropathies and highlight the advantages of WES over single-gene analysis in such cases.

## Case presentation

A 10-year-old Arabic boy was referred to the rheumatology clinic with a presumptive diagnosis of JIA. He was previously healthy up to seven years of age when he presented with arthralgia, swollen joints, and a decreased range of motion in his proximal and distal interphalangeal joints. He also had morning stiffness lasting up to one hour that had partially improved with naproxen. These findings progressed over a few months after onset and extended to the hips, knees, and ankles, and he eventually developed severe valgus deformity of his knees. His medical, prenatal, perinatal, and postnatal histories were unremarkable. There was no history of recurrent infections. His family reported normal vision, hearing, speech, and normal school performance. He underwent extensive evaluation in multiple centers without definitive diagnosis. He was started elsewhere on oral methotrexate once weekly and has been on it for over a year without clinical response.

The family history revealed consanguineous parents. His father is Arabic and has two sisters and two brothers who are all healthy. His mother is Arabic and has one healthy sister and six healthy brothers. She had six pregnancies, and the proband is the youngest of his siblings. The first child died in infancy from birth complications, and the second pregnancy ended in miscarriage. The third and fifth pregnancy outcomes were a healthy female and male, currently aged 21 and 16 years, respectively. The fourth pregnancy outcome was a female currently aged 18 years old with a similar arthritic phenotype. The maternal grandmother and paternal grandmother are siblings, and their parents are consanguineous. They have a sister who had six offspring; four of them have debilitating arthropathy of unknown etiology with severe disability.

**Fig. 1:**
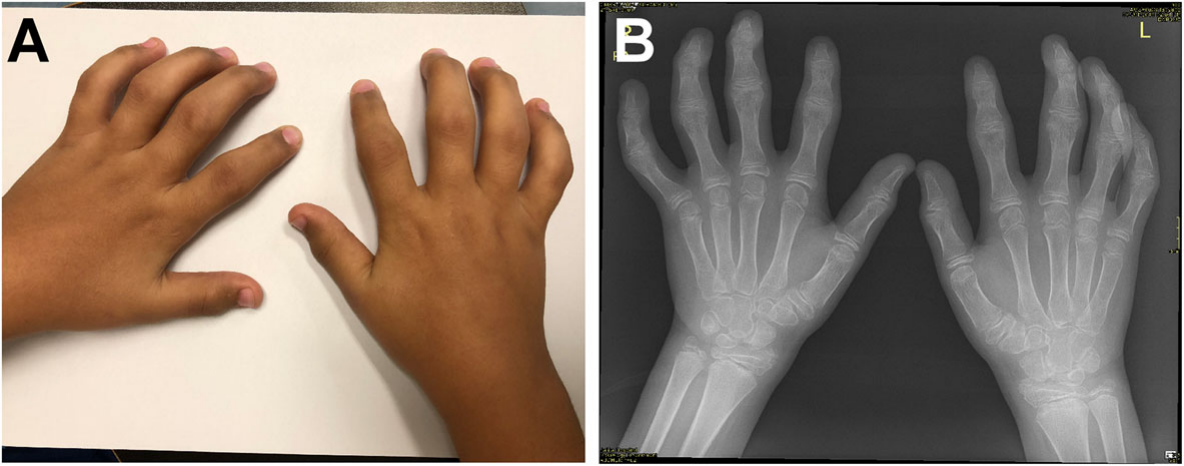
**a**: Swollen wrists and small joints including the proximal interphalangeal joints (PIPs) and distal interphalangeal joints (DIPs) with bony enlargement, tenderness, and fixed flexion deformities. **b**: Radiograph shows arthritic changes involving the interphalangeal joints, more in the PIPs associated with enlarged proximal phalangeal epiphyses and metaphyses. The intercarpal joints and wrist joints showed the same osteoarthritic changes

On physical examination, the proband had normal vital signs. His height was at the 10th percentile, and his weight was at the 75th percentile. His cardiovascular, chest, abdominal, and integumentary examinations were unremarkable. Additionally, his neurological examination was unremarkable, his muscle strength was normal, and his temporomandibular joint was normal. The upper extremity evaluation revealed decreased internal/external rotation range of motion of the shoulders and decreased range of motion with swelling, pain, limitation of the elbows and restriction of pronation and supination of the forearms. The hands and fingers are shown in Fig. 1a. The lower extremity evaluation revealed a severely restricted range of motion of the hips and restricted range of motion of both knees and ankles with swelling and deformities. His toes were deformed and swollen with restricted range of motion. He had no sacroiliac joint tenderness. He had a lordotic posture and decreased range of motion of the cervical spine. He also had a waddling gait.

**Fig. 2:**
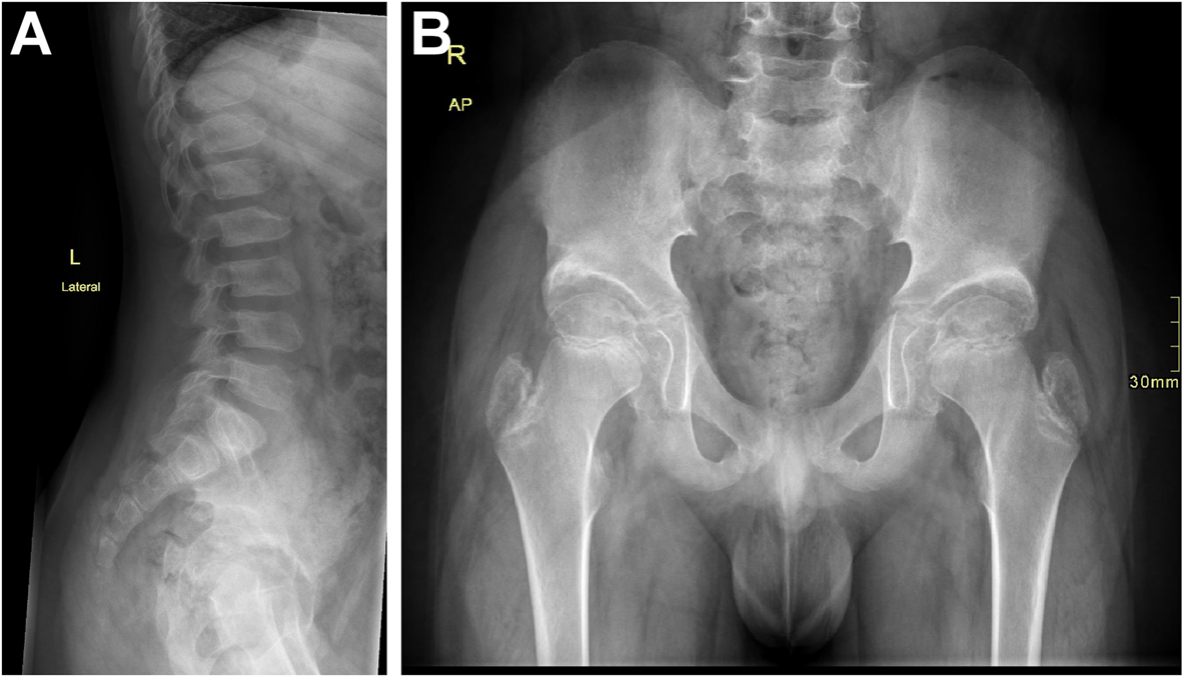
**a**: Spine radiograph showing generalized decreased bone density and flat vertebral bodies with multiple anterior beaking. **b**: There was involvement of both hip joints, with enlarged femoral epiphyses and narrowing of the hip joint space. Medial femoral flattening of the femoral heads with a short femoral neck causing bilateral coca valga was also noted. Marked arthritic changes were present affecting both sides of each joint, with subcortical erosion, subcortical cysts, and narrowing of the joint spaces

**Fig. 3:**
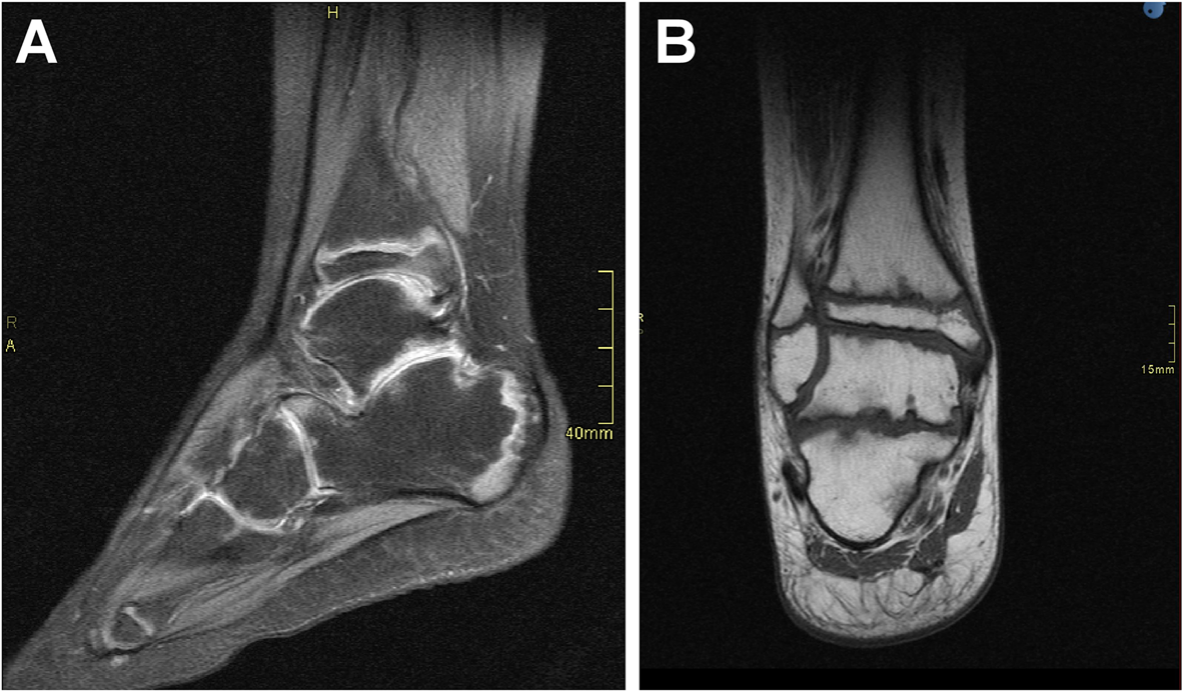
MRI scans performed elsewhere for the ankles (**a** and **b**) revealed the presence of arthritic changes in the form of irregularity in opposing joint surfaces, erosions, and narrow joint spaces with patchy areas of bone marrow edema

The evaluation in our hospital revealed an unremarkable complete blood count, erythrocyte sedimentation rate (ESR), and C-reactive protein (CRP) level as well as normal tests for liver and kidney functions. The antinuclear antibody (ANA), rheumatoid factor, anti-CCP, and HLA-B27 tests were negative. Immunoglobulin IgA, IgM, and IgG levels were normal. Chest radiographs, detailed ophthalmology exams, and cardiology evaluations, including electrocardiography and echocardiography exams, were all normal. The skeletal survey and imaging findings were consistent with PPRD (Fig. 1b, Figs. 2, and 3). Genetic testing was performed at GeneDx laboratory, USA (www.genedx.com). DNA was extracted from the peripheral blood obtained from the proband, mother, father, healthy brother, and an affected sister. The entire exomes of all five family members were captured and sequenced on an Illumina (San Diego, USA) platform. Paired end reads were aligned to the human genome (build GRCh37/hg19), and variants were called, annotated, and filtered using a custom-made pipeline. Segregation analysis was performed to establish a diagnosis in the proband and, possibly, the affected sister. The proband and affected sister carried a novel homozygous frameshift pathogenic variant (c.707delG; p.Ser236Thrfs*5) in the *WISP3* gene (NM_003880.3). The parents were heterozygous, while the unaffected brother did not carry this variant. This variant causes a frameshift starting at position 236 and introduces a premature stop codon 5 amino acids downstream. This is then expected to lead to a truncated or absent protein. Bi-allelic loss of function of the *WISP3* gene is an established disease mechanism in PPRD (OMIM #208230).

Interestingly, the affected sister was also homozygous for a pathogenic missense variant (c.2230G > T; p.Ala744Ser) in the *MEFV* gene (NM_000243.2), while the mother, father, and proband were all heterozygous for the same variant. The unaffected brother did not carry this variant. The proband and the affected sister do not have any noticeable clinical phenotype for familial Mediterranean fever (FMF).

## Discussion

Genetic musculoskeletal disorders with polyarthropathy should be considered in the differential diagnosis of poly-JIA [2]. PPRD, for example, is characterized by progressive arthropathy involving the small peripheral joints in early childhood with progressive stiffness and flexion contractions of the interphalangeal joints associated with metaphyseal bony overgrowth of the metacarpals and phalanges. These features can mimic poly-JIA, leading to a delayed diagnosis. Family history, such as consanguinity and other affected family members, and imaging findings in particular provide important clues for considering early genetic testing. In our case, multiple family members had severe progressive and disabling arthropathy in an inheritance pattern suggestive of an autosomal recessive disorder. Furthermore, skeletal findings on plain joint radiographs and axial skeleton involvement, such as flat vertebral bodies with multiple anterior beaking, kyphoscoliosis, and hyperlordosis, all of which are not typically seen in poly-JIA, were additional clues that favored genetic testing for an accurate diagnosis.

PPRD is a rare autosomal recessive skeletal disorder frequently diagnosed among Arabs [6]; it is attributed to bi-allelic loss of function in the *WISP3* gene, which is essential for the normal growth and function of joint cartilage [3]. Patients with PPRD typically present in early childhood with progressive stiffness and flexion contractions of the interphalangeal joints associated with metaphyseal bony overgrowth of the metacarpals and phalanges mimicking JIA. Eventually, other joints become involved and patients develop disproportionate short stature, abnormal posture, kyphoscoliosis, hyperlordosis, and abnormal gait. The levels of inflammatory markers are within the normal range in these cases, and radiological findings show dysplastic rather than arthritic changes; the characteristic findings include epimetaphyseal expansion and platyspondyly [2, 3]. Unfortunately, there is no known treatment for this condition; patients require supportive care for osteoarthritis-like pain due to joint degeneration and bony dysplasia. Although our initial suspicion was PPRD, we decided to proceed with WES rather than *WISP3* single-gene analysis since a number of rare familial noninflammatory arthropathies should be considered in the differential diagnosis of PPRD [2, 7, 8]. Camptodactyly-arthropathy-coxa vara-pericarditis syndrome (CAPC), for example, is a rare autosomal inherited disorder caused by mutations in the proteoglycan 4 gene (*PRG4*) gene. Clinical manifestations usually start in infancy as camptodactyly of the fifth fingers followed by swelling of interphalangeal joints, wrists, and knees in early childhood, and patients may be diagnosed inaccurately with JIA. Patients usually develop femoral dysplasia and progressive coxa vara. The arthropathy is associated with limited motion with synovial thickening and effusion but without other clinical features of arthritis such as warmth, redness, or tenderness. Some patients may present with pericarditis, sometimes with effusion requiring pericardiocentesis. Synovial tissue biopsy shows proliferative synovium with hypercellularity by infiltrating macrophages with a contribution from proliferating fibroblastic synoviocytes [2, 7].

Inherited idiopathic osteolysis syndromes is another group of rare heterogeneous disorders characterized by destruction and resorption of affected bones. Multicentric osteolysis is notable for interphalangeal joint erosions that mimic severe JIA. Some patients present with nodulosis, arthropathy, and osteolysis (NAO syndrome) as a result of a mutation in the matrix metalloproteinase 2 (*MMP2*) gene. The affected patients are young children presenting with restricted movements of the wrist and ankle joints mimicking arthritis but with rapid progressive resorption of the carpal and tarsal bones resulting in severe deformities and functional disabilities. Inflammatory markers including ESR and CRP level are typically within normal limits, and plain radiography of the hands and feet reveals early destructive osteolytic changes of the carpal and tarsal bones. Other joints can be involved including the elbows, knees, and hips [8, 9]. There is no effective treatment available for these inherited disorders. Therefore, establishing an accurate diagnosis is essential to avoid the use of methotrexate and biologics.

WES in this family led to a timely diagnosis after referral to rheumatology service, which could have been delayed further if a sequential single-gene testing approach was adopted. The WES approach is also considered more cost effective than a stepwise approach. Moreover, it was important to proceed with variant segregation analysis given the family history. The proband and affected sister carried a novel homozygous frameshift pathogenic variant (c.707delG; p.Ser236Thrfs*5) in the *WISP3* gene. Although the observed c.707delG variant has not been identified in large population cohorts, it is interpreted as a pathogenic variant because the homozygous presence of the c.707delG variant is consistent with the classic clinical phenotype in both affected siblings. There have been nearly seventy loss-of-function variants in *WISP3* reported to date in several hundreds of individuals with PPRD of diverse ethnic origins, including frameshifts, deletions and nonsense mutations, but these did not show clear genotype-phenotype correlations [10]. The clinical and radiological phenotype in our case is similar to that of previously reported cases.

In addition to the advantages of WES over single-gene analysis, families are informed that WES can lead to the unexpected detection of variants in other unsuspected genes. In this family, an unanticipated observation was the coexistence of another genetic disorder, which may cause added morbidity in affected individuals in the future. WES detected an unexpected p.A744S *MEFV* variant in the proband, parents, and affected sister that has been reported as a common pathogenic variant in multiple individuals affected with FMF [11]. To date, this additional finding has not had any apparent impact on the clinical phenotype of the arthropathy. However, we will follow the proband closely for any new manifestations related to FMF. We also referred the father and sister to the adult rheumatology department for further evaluation, as the clinical phenotype could have been either subtle or overlooked. Coexisting genetic disorders have been reported in Arabic families [12, 13], and WES has the advantage of detecting more than one disease, whether it has overlapping clinical features or independent clinical manifestations. WES has a high diagnostic yield in the setting of Mendelian disorders and is particularly helpful diagnostically in the highly consanguineous Middle Eastern population including the United Arab Emirates [13, 14].

## Conclusions

The differentiation of poly-JIA from PPRD is challenging, especially in the early phase of PPRD given the polyarticular nature of the disease, prolonged morning stiffness in some patients and the presence of swollen joints with effusions. Both entities present with arthropathy involving the small joints in the hands and feet. Family history, imaging findings, and genetic testing are important tools to help establish the diagnosis and avoid unnecessary use of immunosuppressive medications. WES is superior to single-gene analysis when differential diagnosis is inclusive of more than one inherited disorder, and WES is capable of detecting coexisting diseases.

## Data Availability

Data sharing was not applicable to this article, as no datasets were generated or analyzed during the current study.

## Abbreviations

PPRD: Progressive pseudorheumatoid dysplasia
JIA: Juvenile idiopathic arthritis
WES: Whole-exome sequencing
